# Passive hydrotherapy preserves cartilage and muscle integrity in a murine osteoarthritis model: potential role of integrin αV/TGF-β mechanotransduction

**DOI:** 10.3389/fphys.2025.1633618

**Published:** 2025-10-29

**Authors:** Wu Di, Wang Songyang, Feng Ruibing, Huang Yong, Hu Hao, Duan Xiaofeng, Rong Yang, Dong Yunxiang, Wu Gang

**Affiliations:** ^1^ Department of Orthopedic, The Second People’s Hospital of China Three Gorges University, Yichang, Hubei, China; ^2^ Hubei Provincial Hospital of Traditional Chinese Medicine, Affiliated Hospital of Hubei University of Chinese Medicine, Wuhan, Hubei, China; ^3^ Department of The First Clinical College, Hubei University of Chinese Medicine, Wuhan, Hubei, China; ^4^ School of Sports Medicine, Wuhan Institute of Physical Education, Wuhan, Hubei, China

**Keywords:** osteoarthritis, cartilage, hydrotherapy, swimming, integrin, TGF-β

## Abstract

**Background:**

Osteoarthritis (OA) is a prevalent degenerative joint disease lacking curative treatments. While moderate physical activity such as swimming has been demonstrated to decelerate disease progression, its applicability is limited for individuals unable to perform high-impact or weight-bearing exercises. This study aimed to evaluate whether hydrotherapy—a low-impact, aquatic-based intervention—exerts joint-protective effects comparable to those of swimming in a murine model of OA.

**Methods:**

Male C57BL/6 mice were subjected to destabilization of the medial meniscus (DMM) surgery in the right knee to induce OA and were subsequently randomized into three groups (n = 16 per group): DMM group (control), Hydrotherapy, and Swimming. An additional sham-operated group (n = 16) was included for baseline comparisons. Mice in the intervention groups underwent respective exercise regimens (30 min/session, twice daily, 5 days/week) for 4 or 8 weeks. Histopathological analyses were performed to assess degenerative changes in subchondral bone, articular cartilage, and quadriceps muscle. Additionally, expression levels of key proteins involved in mechanotransduction and tissue remodeling were quantified.

**Results:**

DMM surgery resulted in marked subchondral bone degeneration, cartilage matrix disruption, and quadriceps muscle atrophy. Neither hydrotherapy nor swimming attenuated subchondral bone degeneration. Both interventions mitigated muscle atrophy, potentially via modulation of integrin β1 signaling. Furthermore, hydrotherapy and swimming effectively preserved cartilage structure and suppressed extracellular matrix degradation. These chondroprotective effects are consistent with a reduction in peak joint loading during aquatic exposure and an associated attenuation of integrin αV and TGF-β/SMAD2/3 signaling, although causality was not directly tested in this study.

**Conclusion:**

These findings demonstrate that hydrotherapy confers cartilage-protective benefits comparable to swimming in a mouse model of OA. Possibly through the modulation of mechanosensitive signaling pathways, hydrotherapy may represent a viable, non-pharmacological strategy for delaying OA progression, particularly in individuals with limited capacity for conventional physical exercise.

## 1 Introduction

Osteoarthritis (OA) is one of the most prevalent musculoskeletal disorders globally, affecting over 250 million individuals and imposing a substantial burden on public health systems (Heidari, 2011; Abi et al., 2023). It is characterized by degeneration of articular cartilage, subchondral bone sclerosis, marginal osteophyte formation and synovial inflammation, which together lead to chronic pain, joint stiffness, and functional disability (Kean et al., 2004; Pitsillides and Beier, 2011). The pathogenesis of OA involves the whole joint organ; articular cartilage breakdown is accompanied by alterations in subchondral bone and periarticular tissues in a vicious cycle of degeneration (Man and Mologhianu, 2014). Despite the high disease burden, there are currently no disease-modifying OA drugs, and treatment is largely symptomatic, focusing on pain relief and improved mobility (Zheng et al., 2024). Therefore, non-pharmacological interventions such as exercise therapy have become critical for managing OA and potentially slowing its progression (Filardo et al., 2016).

Mechanical factors play a crucial role in OA development and progression. Under normal conditions, moderate mechanical loading of joints is essential for maintaining cartilage homeostasis and subchondral bone structure (Sun, 2010; Chen et al., 2024). In contrast, abnormal or excessive mechanical stress can disrupt tissue homeostasis and initiate degenerative changes (Serra and Soler, 2019; Filho et al., 2021; Da Silva et al., 2023). Chondrocytes sense and respond to mechanical cues through mechanotransduction pathways, notably via integrin receptors that connect the extracellular matrix (ECM) to the cytoskeleton (Gilbert et al., 2021). Integrins are transmembrane heterodimers that mediate cell–ECM adhesion, transmit mechanical signals, and regulate cellular processes critical for cartilage integrity (Munger and Sheppard, 2011; Wang et al., 2019). In articular cartilage, several integrins such as α5β1, αVβ3, αVβ5, αVβ6, are expressed and serve as mechanosensors linking ECM mechanics to chondrocyte gene expression (Jin et al., 2021; Charlier et al., 2020). Integrin signaling influences the balance of anabolic and catabolic activities in the joint (Li et al., 2017). The integrin αV subunit, in particular, has emerged as a key mediator of pathologic mechanotransduction in OA (Han, 2022). Under high mechanical stress, αV-containing integrins become activated, leading to excessive activation of latent transforming growth factor-β (TGF-β) in cartilage and subchondral bone, and this aberrant TGF-β signaling induces downstream catabolic changes that drive cartilage breakdown (Zhen and Cao, 2014; Zhen et al., 2021). Notably, recent studies demonstrated that chondrocyte-specific deletion of integrin αV reverses abnormal TGF-β activation and attenuates OA cartilage degeneration (Zhen et al., 2021). Conversely, activating αVβ3/αVβ5 integrins on chondrocytes can trigger production of inflammatory cytokines and MMP-13 (Hirose et al., 2020), underscoring the central role of integrin αV/TGF-β signaling in OA pathogenesis. This “double-edged sword” nature of mechanical signaling–beneficial when properly balanced, deleterious when excessive or absent–underpins the rationale for therapeutic exercise in OA.

While excessive mechanical loading is deleterious, therapeutic mechanical modulation can be beneficial for OA. Exercise is known to strengthen periarticular muscles, improve joint function, and potentially slow OA progression. In particular, aquatic exercise is recommended for OA patients because water’s buoyancy reduces joint load while allowing movement. Clinical evidence indicates that aquatic sports improve pain and mobility in knee OA (Hinman et al., 2007; Bartels et al., 2016). Swimming exercise in animal models has also been shown to mitigate OA-related cartilage damage and inflammation (Xue et al., 2022). In addition to active exercise, passive aquatic therapies such as balneotherapy (thermal mineral baths) (Jackson, 1990), or warm-water immersion (hydrotherapy) have long been used to treat arthritis (CURRENCE, 1950; Lei et al., 2024). These benefits are often attributed to improved blood flow, muscle relaxation, and the activation of mechanosensitive pathways by hydrostatic pressure and heat.

However, the mechanistic effects of passive hydrotherapy on joint tissues remain less defined compared to active exercise. It is unclear whether the absence of active movement in warm-water therapy diminishes its chondroprotective efficacy or if thermal and hydrostatic stimuli alone can favorably modulate joint biology. In a DMM mouse model, both forced swimming exercise and limb immobilization during the early OA stage similarly attenuated cartilage degeneration (Xue et al., 2022). The authors postulated that reduced mechanical stimuli lowered the production of catabolic mediators, partially sparing the cartilage. This counterintuitive result raises the question of whether a gentle, passive intervention might achieve benefits comparable to exercise by alleviating aberrant mechanotransduction. Warm-water immersion could potentially create a low-stress biomechanical environment that suppresses deleterious integrin-mediated signaling. Additionally, passive heat exposure is emerging as an “exercise mimetic” that can induce some physiological benefits of exercise. Repeated passive heat therapy has been shown to improve vascular function in patients with limited mobility (Roxburgh et al., 2023), and to promote skeletal muscle hypertrophy and strength in both animals and humans, heat stress elevates tissue metabolism and can activate heat-shock pathways that protect muscle and cartilage cells from stress-related damage (Zanoli et al., 2024). In models of muscle wasting, local heat therapy significantly attenuated muscle atrophy and upregulated hypertrophic signaling proteins, even in the absence of exercise (AlSabagh et al., 2022).

Given this background, we hypothesized that passive warm-water immersion might confer protective effects on the osteoarthritic joint, by reducing abnormal mechanical stress and modulating mechanotransductive pathways. To test this, we used the murine DMM model of knee OA and implemented two interventions–swimming and hydrotherapy–under identical thermal and temporal conditions. Both interventions were applied early in the disease course to target the mechanobiological cascades driving OA progression. We focused on evaluating changes in subchondral bone architecture, articular cartilage integrity, cartilage matrix composition, and quadriceps muscle morphology. We also examined the expression of integrins in articular cartilage, which are highly correlated with mechanical stress, to elucidate the molecular mechanisms. Our purpose was to determine whether a passive aquatic therapy (hydrotherapy), which imparts warmth and buoyancy without active joint movement, can achieve similar chondroprotective and anabolic effects as active swimming exercise. Demonstrating equivalence would highlight integrin-mediated mechanotransduction as a key therapeutic target and support the use of hydrotherapy as a low-impact treatment for OA, particularly for individuals who cannot tolerate strenuous exercise.

## 2 Methods

### 2.1 Animals

64 male 8-week-old C57BL/6 mice (weighing 20–25 g) were procured from the Hunan SJA Laboratory Animal Co., Ltd. and kept under specific pathogen-free conditions in standard laboratory settings: 25 °C ± 2 °C, 50% ± 5% humidity, and a 12-h light/dark cycle, with *ad libitum* access to food and water. After 1 week of acclimatization, 16 mice were randomly assigned to the sham group, while the remaining 48 mice underwent DMM surgery under anesthesia induced by intraperitoneal injection of sodium pentobarbital (50 mg/kg, Lulong Biotechnology Co., Ltd., China). All surgeries were performed under aseptic conditions. For the DMM procedure, the right knee joint was opened and the medial meniscotibial ligament (MMTL) of the medial meniscus (which anchors the cranial horn of the medial meniscus to the anterior tibial plateau) was identified and carefully transected, while leaving the medial meniscus and other ligaments intact (Glasson et al., 2007).In the Sham group, the joint capsule was opened and the MMTL was exposed but not cut (i.e., the ligament was left intact). After surgery, the incision was closed and treated daily with an iodophor antiseptic solution to prevent infection.

### 2.2 Experimental design

48 mice underwent DMM surgery on the right knee to induce OA. One week post-surgery, mice were randomly assigned into DMM groups (no exercise intervention), Swimming group and Hydrotherapy group (n = 16 per group). Mice in DMM group were allowed unrestricted movement in their cages with standard chow and water but received no additional interventions. Mice in Swimming group underwent swimming exercise intervention in a water tank (50 cm*60 cm*100 cm) maintained at 37 °C ± 2 °C, verified before and after each session with a digital probe. This temperature was selected to minimize cold-stress in mice and to mirror clinically used warm-water protocols in rehabilitation contexts. No additional thermal manipulation was applied, and intra-articular temperature was not instrumented in this study. Each session consisted of free swimming for 30 min, performed twice daily with at least 6 h of rest between sessions, 5 days per week.

**FIGURE 1:**
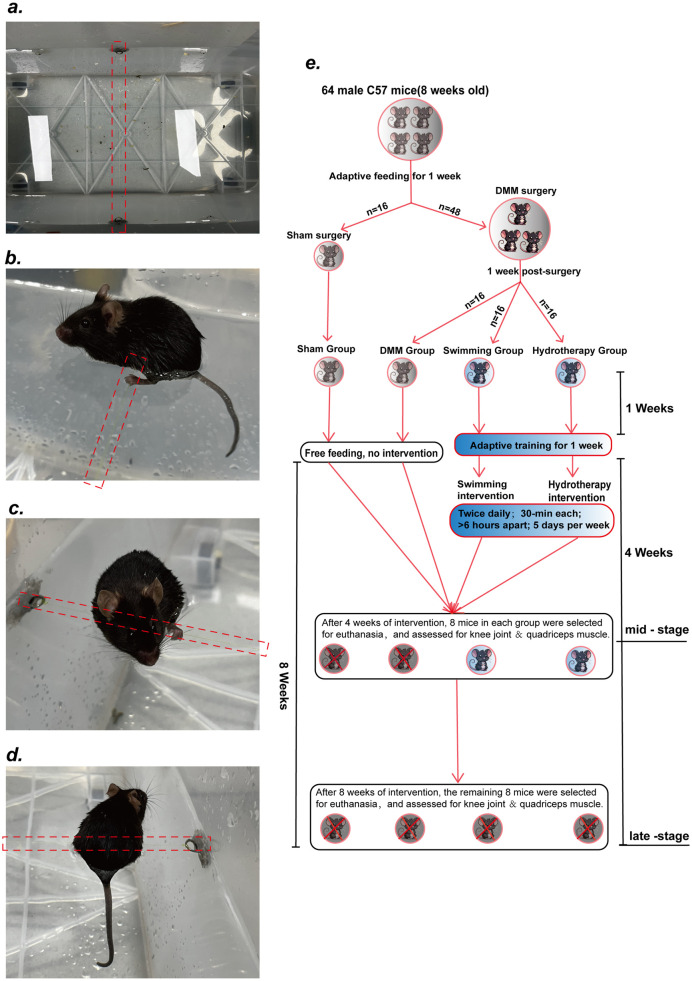
Hydrotherapy Protocol and Experimental Design. **(a)** Suspend a thin nylon thread about 1.5 cm below the surface of the water in the tank. **(b–d)** After entering the water, the mice were able to grasp the nylon thread voluntarily, maintaining a stationary floating posture with their hind limbs fully submerged. **(e)** Diagram of Experimental design.

Mice in Hydrotherapy group underwent static hydrotherapy intervention in the same water conditions (37 °C ± 2 °C). A thin nylon thread was suspended ∼1.5 cm below the water surface of the tank (Figure 1a). Mice placed in the warm water naturally grasped the thread and maintained a stationary floating posture with hind limbs submerged (Figures 1b–d). Occasional brief swimming occurred but mice quickly resumed floating. The hydrotherapy sessions were matched in frequency and duration to the swimming sessions.

To ensure adaptation to the intervention, all mice underwent a 1-week pre-experimental acclimation period during which intervention durations were gradually increased. By the end of this period, mice in the Swimming group could swim continuously for 30 min without signs of stress, and mice in the Hydrotherapy group could grasp the thread and maintain a stable floating position without external stimulation. Half of the mice from each group were euthanized at week 4 (mid-stage OA), and the remaining half at week 8 (late-stage OA). Right knee joint tissues and quadriceps muscles were collected for further histological and molecular analyses (Figure 1e).

It should be pointed out that we did not perform quantitative home-cage activity tracking; therefore, spontaneous activity between sessions was not directly measured. These design features minimize, but do not eliminate, the possibility that differences in between-session activity contributed to group differences.

### 2.3 Micro-computed tomography (μCT) scanning

The right knee joint of the mice were collected, excess soft tissue was removed, and specimens were fixed in 4% Paraformaldehyde (G1101-500ML, Servicebio, China) for 24 h. Bone tissue evaluation was performed using a Skyscan 1276 μCT instrument (Bruker micro-CT, Kontich, Belgium) with 85 kV, 200 μA and10 μm resolution. Images were reconstructed using NRecon software (Bruker micro-CT, Kontich, Belgium), and a fine region of interest (ROI) was generated. Bone parameters within this ROI were determined using a constant threshold (80–255) for trabecular binarization. Structural parameters were further analyzed using the CTAn program (Bruker, Karlsruhe, Belgium). These parameters included the number and volume of osteophytes, the number of ectopic calcifications, bone surface area/bone volume (BS/BV, 1/mm), bone volume/total volume (BV/TV, %) and structure model index (SMI).

### 2.4 Histological assessment

Knee-joint and quadriceps femoris samples were fixed in 4% paraformaldehyde solution. The knee-joint samples tissue was decalcified with 10% ethylenediaminetetraacetic acid (G1105-500 ML–100ML, Servicebio, China) solution for 1 week. Then, the knee-joint and quadriceps femoris tissues were dehydrated, cleaned, and embedded in paraffin. Paraffin-embedded samples were cut into 5 µm thick tissue sections using a tissue slicer. Sections of knee joint tissue were stained with Safranin O-Fast Green (G1053-100ML, Servicebio, China). Sections of quadriceps femoris tissue were stained with hematoxylin and eosin (H&E) (G1005-100ML, Servicebio, China).

Articular cartilage in the tibial plateau was evaluated using the modified Mankin score (score: 0–15), which is a histological scoring system for articular cartilage quality using sagittal sections. Quadriceps femoris muscle cross-sectional area (CSA) was measured by staining with H&E. H&E sections were imaged at ×10 magnification. Microscopic observation and image collection were performed using a Leica DMI6000 B microscope (Leica Microsystems GmbH). More than 100 randomly selected fibres from each muscle were used to measure muscle CSA using ImageJ software (version 1.53).

### 2.5 Western blot analysis

To collect the articular cartilage, mice were anesthetized and placed on a cold platform for dissection under a stereomicroscope. After shaving and disinfecting the right hind limb, a small incision was made to expose the knee joint. The quadriceps femoris was carefully removed and transferred to a 4 °C pre-cooled centrifuge tube. Surrounding tissues were meticulously removed to expose the joint surfaces. The dissection was performed with fine micro-surgical instruments to ensure precision. Only the articular cartilage on the femoral condyles and tibial plateau was retained. A disposable micro-scalpel was then used to gently scrape a thin layer of cartilage from these surfaces, ensuring that no subchondral bone or residual synovial tissue was included to avoid contamination of the cartilage sample. Knee cartilage or quadriceps femoris were homogenized in RIPA buffer (G2002-30ML, Servicebio, China) supplemented with protease inhibitor PMSF(G2008-1ML, Servicebio, China) and centrifuged at 12,000 × g for 10 min at 4 °C. The supernatant containing protein extracts was collected, and the protein concentration was determined using a protein assay kit (G.26–200T, Servicebio, China). Equal amounts of proteins were separated by 12% sodium dodecyl sulfate–polyacrylamide gel electrophoresis (SDS-PAGE) and transferred onto polyvinylidene difluoride (PVDF) membranes. The PVDF membranes were blocked with TBST (Tris-buffered saline with Tween) containing 5% bovine serum albumin for 1 h at room temperature. Primary antibodies were diluted in TBST and incubated with the membranes overnight at 4 °C (Antibody integrin β1, 1:1000, 4706S; integrin α5, 1:1000, 4705T; integrin αV, 1:1000, 4711T, CST, United States of America. Antibody integrin α7, 1:2500, ab203254, ABCAM, United States of America. Antibody TGF-β, 1:1000, P20929; SMAD2/3, 1:1000, P20682; p-SMAD2, 1:1000, P20251; p-SMAD3, 1:1000, P20278, ProMadb, China. Antibody MMP13, 1:2000, 18165-1-AP; Collagen II, 1:2000, 28459-1-AP; Aggrecan, 1:1000, 68350-1-Ig; β-actin, 1:20000, 66009-1-Ig; gapdh, 1:10000, 10494-1-AP, Proteintech, China). Subsequently, the membranes were incubated with secondary antibody (Goat Anti-Rabbit, 1:10000, SA00001-2; Goat Anti-Mouse, 1:10000, SA00001-1, Proteintech, China) at room temperature for 60 min and washed three times with TBST for 5 min each. The blot proteins were visualized using an enhanced chemiluminescence (ECL) detection kit. ImageJ (version 1.53) software was employed to analyze the optical density of the target bands and calculate the relative expression of each protein.

### 2.6 Immunohistochemical analysis

Paraffin sections of mouse knee joints were baked, dewaxed and hydrated.Antigen retrieval was conducted using a citrate antigen retrieval solution (G1202, Servicebio, China). Subsequently, the sections were treated with a 3% hydrogen peroxide solution to inhibit endogenous peroxidase activity. Blocking was achieved by incubating the sections with normal goat serum for 30 min at room temperature. Overnight incubation with primary antibodies (Antibody MMP13, 1:100, 18165-1-AP; Collagen II, 1:100, 28459-1-AP; Aggrecan, 1:100, 68350-1-Ig, Proteintech, China) followed. This was succeeded by incubation with Goat anti-Rabbit secondary antibody (1:2500, RGAR011, Proteintech, China) for 60 min at room temperature. The sections were stained with DAB chromogen solution (G1212-200T, Servicebio China), counterstained with hematoxylin, dehydrated using an ethanol gradient, and mounted. Microscopic observation and image collection were performed using a Leica DMI6000 B microscope (Leica Microsystems GmbH). The number of positive cells was quantitatively analyzed using ImageJ software (version 1.53).

### 2.7 Statistical analysis

Statistical analysis was conducted using either a two-tailed unpaired Student’s t-test or ANOVA with Tukey’s *post hoc* test. The analyses were performed with a minimum of three replicates using R. Results are expressed as Mean ± SEM of at least 3 independent experiments. A p-value of less than 0.05 was considered statistically significant.

## 3 Results

### 3.1 Hydrotherapy and swimming do not produce a positive effect on degenerative lesions of the subchondral bone in osteoarthritis of the knee

**FIGURE 2:**
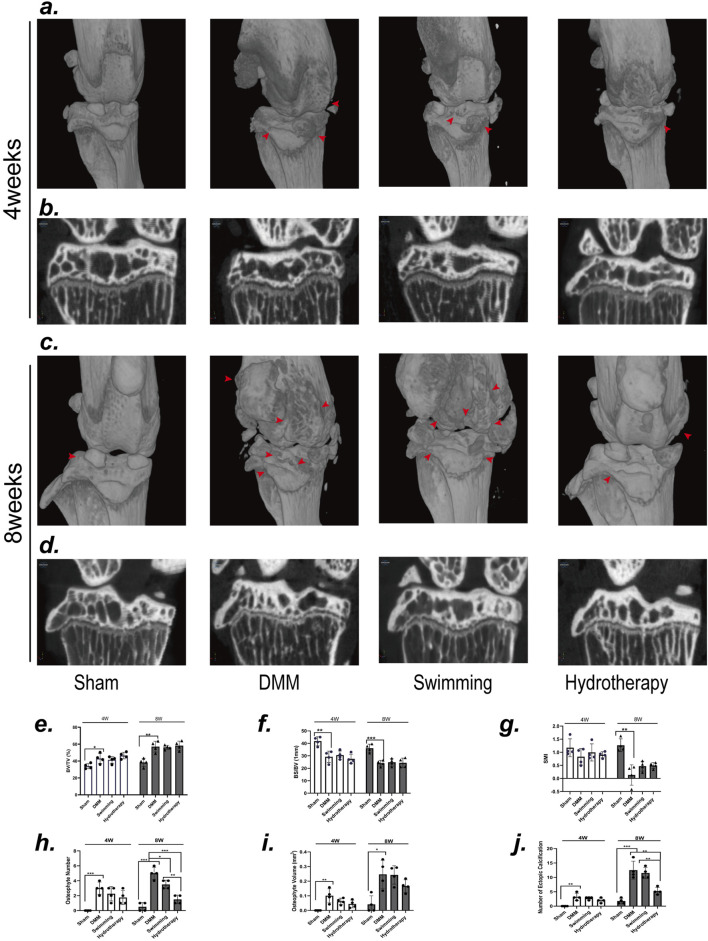
Neither swimming nor hydrotherapy improved DMM-induced degeneration of the subchondral bone in the knee, but hydrotherapy reduces the formation of osteophytes and ectopic calcifications. **(a)** Three-dimensional micro-CT images of the knee joint in each group after 4 weeks of intervention. **(b)** Coronal section of knee subchondral bone in each group after 4 weeks of intervention. **(c)** Three-dimensional micro-CT images of the knee joint in each group after 8 weeks of intervention. **(d)** Coronal section of knee subchondral bone in each group after 8 weeks of intervention. The red arrow points to the osteophyte. **(e,f)** Quantitative analysis of BV/TV, BS/BV, and SMI. DMM induced significant degeneration of the subchondral bone in the knee joint, characterized by an increased BV/TV index, and decreased BS/BV and SMI index. Neither swimming nor hydrotherapy effectively ameliorated the subchondral bone degeneration. **(g–j)** Quantitative analysis of the number of osteophytes, the volume of osteophytes and the number of ectopic calcifications. Hydrotherapy significantly reduced the number of osteophytes and ectopic calcifications, but had no significant effect on the volume of osteophytes. Data are shown as the mean ± SD, n = 4. *p < 0.05, **p < 0.01, ***p < 0.001.

μCT analysis of subchondral bone microarchitecture revealed degenerative changes in the DMM osteoarthritis model. Compared to sham-operated controls, the DMM group exhibited a significantly higher BV/TV and lower BS/BV at mid-stage and late-stage OA (*p* > 0.05), indicating subchondral bone sclerosis. By the late stage, DMM mice also showed a decrease in SMI (*p* > 0.05), reflecting a shift toward plate-like trabecular structure. Neither hydrotherapy nor swimming prevented these microstructural changes, BV/TV, BS/BV, and SMI values in both intervention groups remained similar to those in the DMM group at the corresponding time points (*p* > 0.05) (Figures 2b,d–g).

Notably, swimming and hydrotherapy did not demonstrate a significant therapeutic effect on the degenerative changes in subchondral bone of the knee joint in OA, μCT three-dimensional reconstructions revealed distinct differences in osteophyte and calcified tissue formation among the three groups (Figures 2a,c). At 4 weeks, no significant differences were observed in the number, volume of osteophytes, or the number of ectopic calcifications in the knee joints of the DMM, swimming, and hydrotherapy groups (*p* > 0.05). However, by 8 weeks, the number of osteophytes in the DMM group was significantly higher than in the swimming and hydrotherapy groups (*p* < 0.05), whereas the hydrotherapy group exhibited a significantly lower number of osteophytes compared to the swimming group (*p* < 0.05). In terms of osteophyte volume, no significant differences were observed among the DMM, swimming, and hydrotherapy groups at the 8-week mark (*p* > 0.05). On the other hand, with respect to the number of ectopic calcifications in the knee joints, while no significant difference was observed between the DMM and swimming groups at 8 weeks (*p* > 0.05), the hydrotherapy group had significantly fewer ectopic calcifications compared to both the DMM and swimming groups (*p* < 0.05) (Figures 2h–j). These findings suggest that although swimming and hydrotherapy did not actively prevent the degenerative changes of subchondral bone in OA, the presence of osteophytes and calcified tissue formation displayed notable differences, indicating that the interventions may have some impact on the progression of the disease.

### 3.2 Hydrotherapy and swimming reduce degenerative changes in osteoarthritic cartilage of the knee

Articular cartilage integrity was evaluated by Safranin-O/Fast Green staining, and degeneration was scored using the Mankin scale. By the mid-stage of OA, the DMM group exhibited evident cartilage erosion at the tibial plateau, corresponding to a Mankin’s score indicative of moderate degeneration. At the late stage, cartilage loss in DMM knees was severe, with Mankin’s scores reflecting advanced degeneration and loss of cartilage matrix.

**FIGURE 3:**
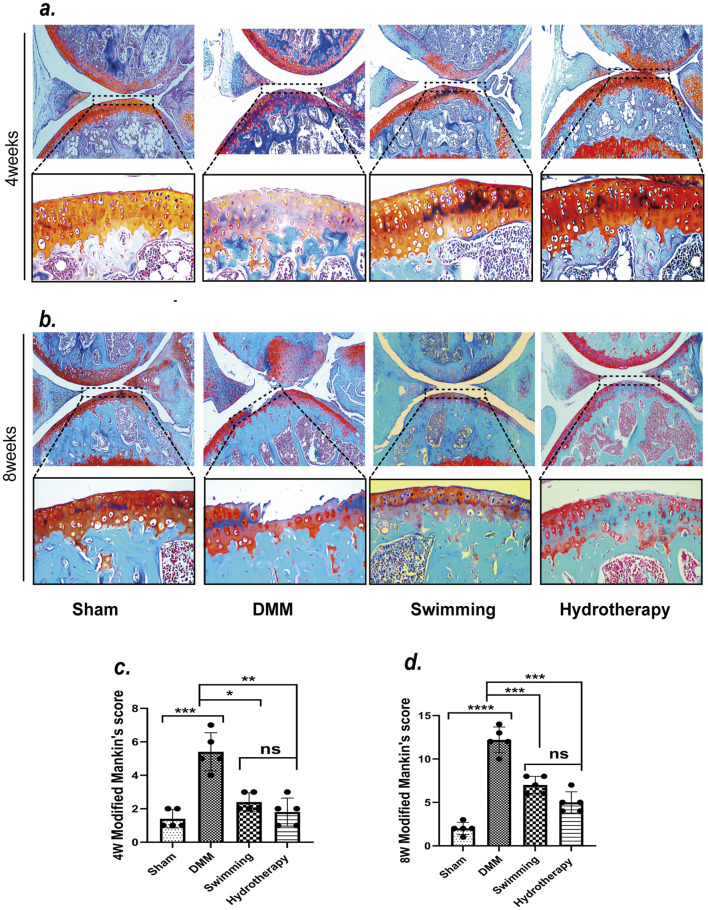
Safranin-O/Fast Green staining showed that swimming and hydrotherapy played a positive role in the cartilage area of the knee. **(a,b)** Safranin-O/Fast Green staining of articular cartilage in each group after 4 and 8 weeks of intervention. **(c,d)** Modified Mankin’s scores for articular cartilage in each group after 4 and 8 weeks of intervention. DMM surgery resulted in significant degeneration of the articular cartilage in the knee joint, as evidenced by a marked increase in Modified Mankin’s score compared to the Sham group. Both swimming and hydrotherapy interventions attenuated cartilage degeneration at 4 and 8 weeks, with significantly lower Modified Mankin’s scores compared to the DMM group. No significant difference in Mankin’s score was observed between the swimming and balneotherapy groups. Data are shown as the mean ± SD, n = 5. *p < 0.05, **p < 0.01, ***p < 0.001, ****p < 0.0001.

Hydrotherapy and swimming both attenuated the progression of cartilage damage. At mid and late stages, mice in the hydrotherapy and swimming groups retained more complete cartilage compared to the untreated DMM group. Accordingly, their Mankin’s scores were significantly lower than those of DMM mice (*p* < 0.05), indicating only mild-to-moderate degeneration. There was no significant difference between the hydrotherapy and swimming groups in cartilage morphology or Mankin’s score (*p* > 0.05), suggesting that both interventions provided similar chondroprotective effects (Figure 3).

### 3.3 Integrin αV-mediated TGF-β signalling pathway may be one of the mechanisms by which swimming and hydrotherapy alleviate cartilage degeneration

**FIGURE 4:**
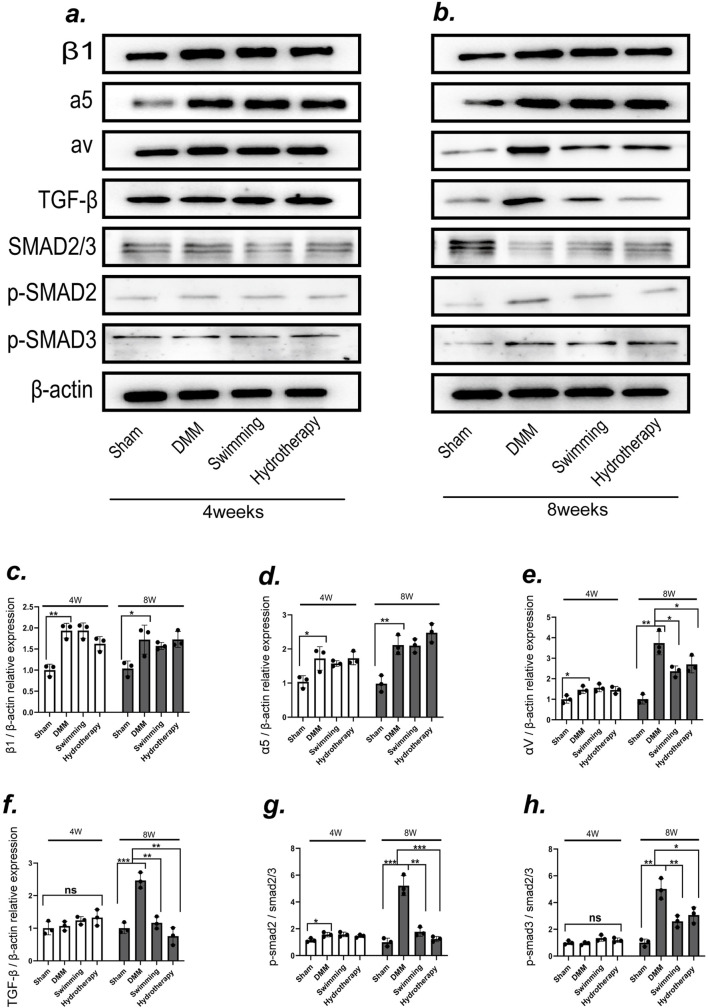
The integrin αV-mediated TGF-β/SMAD2/3 signaling pathway may be one of the mechanisms by which swimming and hydrotherapy alleviate cartilage degeneration. **(a,b)** Protein expression levels of integrins β1, integrins α5, integrins αV, TGF-β, SMAD2/3, P-SMAD2 and P-SMAD3 in the knee cartilage were measured by Western blotting after 4 and 8 weeks of intervention in each group. **(c–e)** The bar graph of the Relative expression of integrins β1, α5 and αV by optical density analysis with reference to β-actin. DMM significantly upregulated the expression of integrins β1, α5, and αV in cartilage. Neither swimming nor hydrotherapy had an effect on the expression of integrins β1 and α5 after 4 or 8 weeks of intervention. However, both interventions significantly reduced the expression of integrin αV after 8 weeks. **(f-h)** The bar graph of the Relative expression of TGF-β, P-SMAD2 and P-SMAD3 by optical density analysis with reference to β-actin or SMAD2/3. Activation of the TGF-β/Smad2/3 signaling pathway was associated with integrin αV expression. DMM significantly upregulated TGF-β expression and activated the phosphorylation of SMAD2 and SMAD3 in knee cartilage. Both swimming and hydrotherapy interventions for 8 weeks reduced TGF-β expression and suppressed SMAD2 and SMAD3 phosphorylation. All data were normalized to the NC group. Data are shown as the mean ± SD, n = 3. *p < 0.05, **p < 0.01, ***p < 0.001.

To explore potential mechanisms of cartilage protection, we examined the expression of integrins associated with mechanotransduction in cartilage via Western blot analysis (Figures 4a,b). In the DMM group, integrin β1 and integrin α5 levels were significantly elevated relative to sham group at both mid and late OA stages (*p* < 0.05). Neither hydrotherapy nor swimming altered this OA-induced upregulation of β1 and α5, their levels in the treated groups remained comparable to those in the untreated DMM group (*p* > 0.05). These results indicate that the interventions did not affect the abnormal increases of integrin β1 and α5 in osteoarthritic cartilage (Figures 4c,d).

In contrast, integrin αV exhibited a different pattern. DMM cartilage showed a significant increase in integrin αV expression at the mid-stage compared to sham, and this level rose further by the late stage (*p* < 0.05). Hydrotherapy and swimming had no effect on αV at mid-stage (*p* > 0.05), but by the late stage both interventions significantly lowered integrin αV levels in cartilage compared to the DMM group (*p* < 0.05). There was no significant difference between the hydrotherapy and swimming groups in αV expression at that time point (*p* > 0.05) (Figure 4e).

Consistent with the changes in integrin αV, activation of its downstream TGF-β signaling pathway in cartilage was modulated by the interventions. In late-stage DMM cartilage, TGF-β expression was high and the downstream mediators SMAD2/3 were heavily phosphorylated (*p* < 0.05), indicating activation of TGF-β/Smad signaling. Hydrotherapy and swimming attenuated this pathway: the treated cartilage showed reduced TGF-β levels and decreased phosphorylation of SMAD2 and SMAD3 compared to DMM (*p* < 0.05). These findings suggest that modulation of the integrin αV–mediated TGF-β/Smad pathway may be one mechanism by which hydrotherapy and swimming ameliorate OA-induced cartilage degeneration (Figures 4f–h). Notably, the divergence in integrin αV and downstream SMAD2/3 phosphorylation emerged at 8 weeks rather than 4 weeks, consistent with a cumulative, threshold-like response to the repeated daily period of buoyant unloading and uniform hydrostatic pressure during immersion.

### 3.4 Hydrotherapy and swimming reduce degenerative changes in the cartilage matrix in osteoarthritis of the knee

**FIGURE 5:**
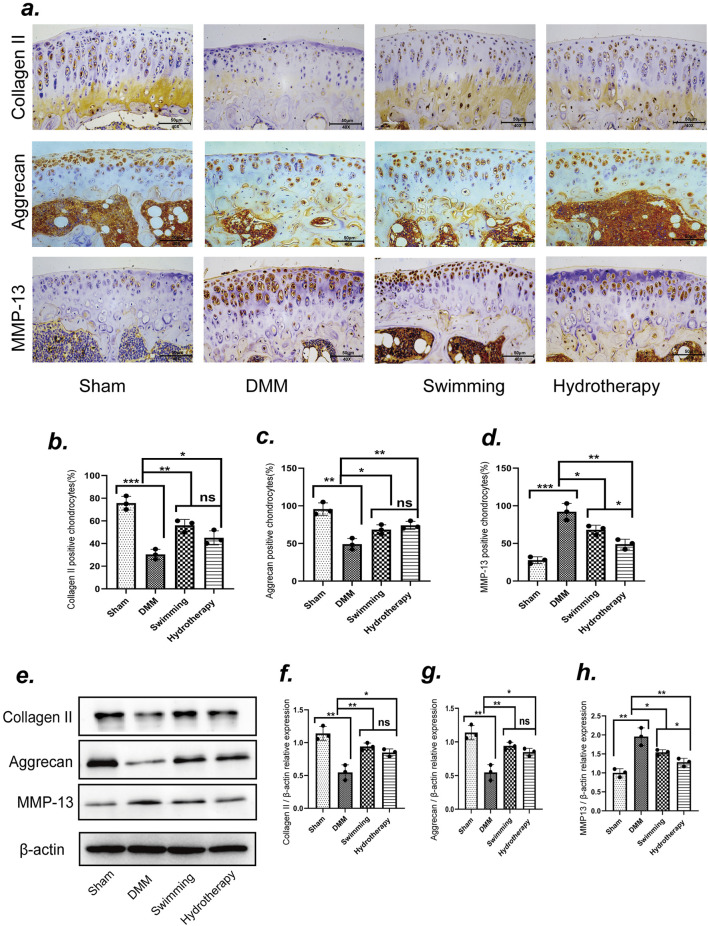
Swimming and hydrotherapy alleviate cartilage matrix degradation. **(a)** Immunoreactivities of Collagen II, Aggrecan and MMP-13 positive chondrocytes in the knee cartilage of the tibial plateau after 8 weeks of intervention in each group. **(b–d)** The number of Collagen II, Aggrecan and MMP-13 positive chondrocytes. **(e)** Protein expression levels of Collagen II, Aggrecan and MMP-13 in the knee cartilage were measured by Western blotting after 8 weeks of intervention in each group. **(f–h)** The bar graph of the Relative expression of CollagenII, Aggrecan and MMP-13 by optical density analysis with reference to β-actin, all data were normalized to the NC group. DMM accelerated cartilage matrix degradation, as evidenced by decreased expression of Collagen II and Aggrecan, and increased expression of MMP-13. Both swimming and hydrotherapy interventions attenuated cartilage matrix degradation. Data are shown as the mean ± SD, n = 3. *p < 0.05, **p < 0.01, ***p < 0.001.

At the late stage of OA, both immunohistochemistry and Western blot analyses demonstrated significant loss of cartilage extracellular matrix components in the DMM group (Figures 5a,e). Specifically, Collagen II and Aggrecan levels in DMM cartilage were markedly lower than in sham controls, while the catabolic enzyme MMP-13 was greatly elevated (*p* < 0.05). Hydrotherapy and swimming preserved these matrix components. The intervention groups showed significantly higher Collagen II and Aggrecan expression in cartilage compared to the DMM group (*p* < 0.05). There was no significant difference between hydrotherapy and swimming in their effects on collagen II or aggrecan levels (*p* > 0.05) (Figures 5b,c,f,g).

Hydrotherapy and swimming also mitigated the excessive increase of MMP-13 in osteoarthritic cartilage. Immunohistochemical staining and Western blot analyses indicated that MMP-13 expression was reduced in both intervention groups, with hydrotherapy showing a more pronounced reduction than swimming (*p* < 0.05). These data suggest that both interventions protect the cartilage matrix by enhancing matrix component retention and reducing catabolic activity, with hydrotherapy potentially having a stronger effect on inhibiting matrix degradation (Figures 5d,h).

### 3.5 Hydrotherapy and swimming improve quadriceps atrophy due to osteoarthritis of the knee

**FIGURE 6:**
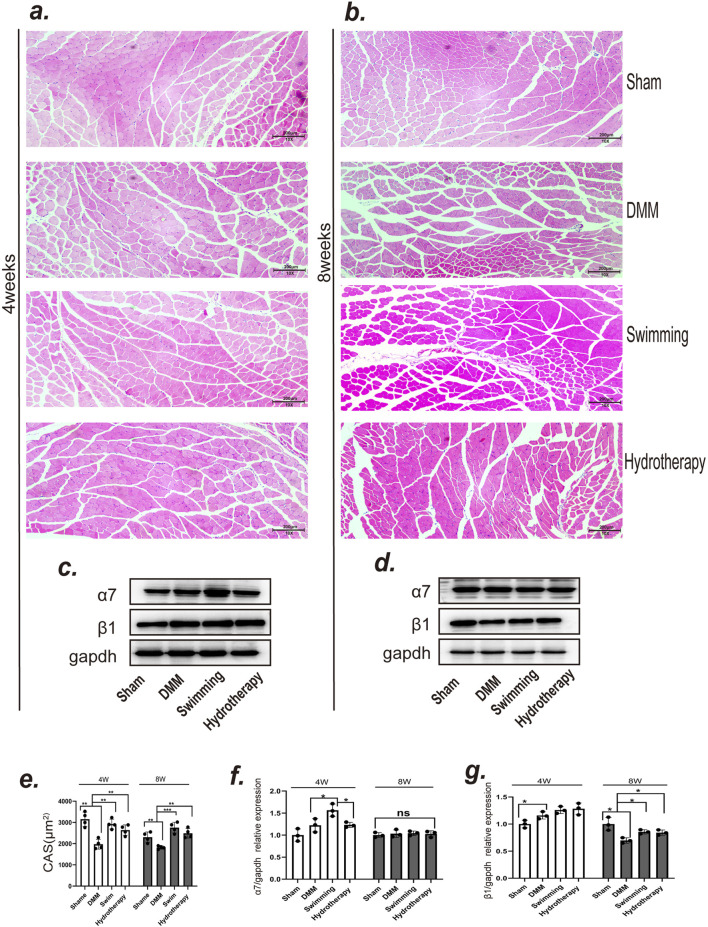
Both swimming and hydrotherapy interventions mitigated quadriceps femoris atrophy. **(a,b)** H&E staining of quadriceps femoris in each group after 4 and 8 weeks of intervention. **(c,d)** Protein expression levels of integrins α7 and β1 in the quadriceps femoris were measured by Western blotting after 4 and 8 weeks of intervention in each group. **(e)** Quantitative histogram of quadriceps femoris fiber CSA. Data are shown as the mean ± SD, n = 4. **(f,g)** Quantitative histogram of integrins α7 and β1 expression levels. Data are shown as the mean ± SD, n = 3. *p < 0.05, **p < 0.01, ***p < 0.001.

Knee OA led to notable atrophy of the quadriceps muscle, and both hydrotherapy and swimming helped counteract this effect. Histological analysis of quadriceps cross-sectional area (CSA) by H&E staining showed that the DMM group had a smaller muscle fiber CSA than the sham group at both mid and late stages (*p* < 0.05), consistent with OA-induced muscle atrophy. Mice in the hydrotherapy and swimming groups, however, maintained significantly larger muscle fiber CSA compared to DMM mice at the same time points (*p* > 0.05), indicating preserved muscle mass. There was no significant difference between hydrotherapy and swimming in their effect on muscle fiber size (*p* > 0.05). These results demonstrate that both interventions ameliorated OA-induced quadriceps muscle atrophy (Figures 6a,b,e).

To investigate the molecular basis of this muscle preservation, we assessed the expression of muscle-related integrins in the quadriceps (Figures 6c,d). Integrin α7 showed no significant difference between DMM and sham groups at mid-stage (*p* > 0.05). Interestingly, the swimming group exhibited a significantly higher integrin α7 level at mid-stage compared to DMM (*p* < 0.05), whereas hydrotherapy had no such effect (*p* > 0.05). By late stage, integrin α7 expression equalized, with no significant differences among sham, DMM, swimming, or hydrotherapy groups (*p* > 0.05) (Figure 6f).

Integrin β1 in muscle showed an opposite trend over time. At mid-stage, integrin β1 was significantly elevated in the DMM group relative to sham (*p* < 0.05), and neither hydrotherapy nor swimming altered this early OA-induced increase (*p* > 0.05). By late stage, integrin β1 in the DMM group had dropped below the level in sham (*p* < 0.05), indicating a loss of this integrin with prolonged OA and muscle atrophy. Notably, both hydrotherapy and swimming maintained higher integrin β1 expression in late-stage muscle compared to the DMM group (*p* < 0.05). These data suggest that hydrotherapy and swimming may help preserve muscle mass in OA at least in part by sustaining integrin β1 expression, thereby promoting muscle fiber integrity and counteracting atrophy (Figure 6g).

## 4 Discussion

In this study, we investigated the effects of two distinct aquatic interventions–active swimming and passive hydrotherapy–on knee osteoarthritis in a DMM mouse model. Our findings demonstrate that passive warm-water immersion without joint movement can produce chondroprotective and anabolic outcomes equivalent to those of active exercise. Both interventions significantly blunted OA progression, as evidenced by preservation of cartilage structure and matrix, and attenuation of quadriceps muscle atrophy. Notably, the molecular analyses revealed a common mechanism: both swimming and hydrotherapy suppressed the integrin αV/TGF-β mechanotransduction pathway in the joint. These results suggest that the therapeutic benefits of aquatic activity in OA may derive largely from the altered mechanical environment and mechanosignaling, rather than the active motion *per se*. To our knowledge, this is the first evidence that a fully passive hydrotherapy regimen can match the efficacy of exercise in preventing OA-related joint degeneration *in vivo*. We originally anticipated that active swimming would confer superior benefits, given the well-documented positive effects of exercise on cartilage nutrition and muscle strengthening. Instead, hydrotherapy–involving no voluntary movement–achieved virtually the same level of cartilage protection and muscle preservation. A key novel finding of our study is the unexpected equivalence between swimming and passive immersion in protecting the osteoarthritic joint.

The plausibility that a 60-min daily intervention modulates αV/TGF-β signaling rests on the non-linear, threshold-sensitive nature of mechanotransduction in OA cartilage. Altering peak and pattern of joint stress—rather than total exposure time—can switch latent TGF-β activation off, thereby reducing downstream catabolic signaling. Daily immersion provides a recurrent period of near-zero ground reaction and shear forces together with uniform hydrostatic pressure and warmth, creating a biomechanical and thermal milieu that repeatedly “resets” pathological signals. The late emergence (8 weeks) of αV/TGF-β attenuation supports a cumulative dosing effect rather than an acute response. This concept aligns with prior work showing that both decreased excessive loading and physiologic static pressure can normalize joint cell signaling. The absence of a subchondral response at 4 weeks is consistent with known temporal dynamics of DMM-induced remodeling, where architectural changes accrue progressively over weeks and are often more resolvable after 8–12 weeks (Botter, 2010). Moreover, while daily warm-water immersion reduces pathological peak loads and normalizes mechanosignaling—thereby protecting cartilage—buoyancy also lowers skeletal strain below classic osteogenic thresholds defined by Frost’s mechanostat, limiting an anabolic response in bone at early/mid OA stages (Frost, 1987). In line with this distinction, athletes engaged in aquatic training typically exhibit lower BMD than weight-bearing athletes, highlighting that aquatic exercise is metabolically active yet mechanically low-strain for bone. Consequently, our null subchondral bone finding at 4 weeks should be interpreted as an expected outcome of a mechanothermal, low-strain intervention rather than a discrepancy with reports demonstrating subchondral bone benefits using higher-strain, land-based exercise or longer exposures.

Interestingly, neither hydrotherapy nor swimming produced a detectable improvement in DMM-induced subchondral bone alterations in our model. We therefore posit a division of labor between tissues: cartilage benefits early from repeated relief of pathological loading and altered αV/TGF-β signaling, whereas subchondral bone would require either longer duration and/or higher-strain modalities to show μCT-level architectural change. This framework integrates our aquatic findings with the broader exercise literature across OA stages. OA is known to involve complex changes in subchondral bone architecture, including early bone loss and later sclerosis, marrow lesions, and osteophyte formation​. These changes are thought to be driven by abnormal mechanical loading and bone remodeling processes that may not be easily reversed over the relatively short duration of our interventions. Our data align with this, as 4–8 weeks of exercise were insufficient to normalize subchondral bone structure once OA was established. Similarly, a previous study reported that swimming exercise had only a mild effect on subchondral bone thickening in mid-stage OA, with no significant impact at later stages (Xue et al., 2022). Thus, while exercise confers clear benefits to cartilage and muscle, its influence on the subchondral bone may require longer treatment or combination therapies to manifest. Notably, we did observe that hydrotherapy-treated mice developed fewer osteophytes and ectopic calcifications in the knee joint compared to the swimming group. The reduction of osteophyte formation by hydrotherapy could be a result of altered mechanical environment around the joint–perhaps reduced peak forces or more stable joint kinematics–thereby decreasing the stimuli for osteophyte development. This finding suggests a slight advantage of hydrotherapy in curbing certain bone-related aspects of OA, though the exact mechanism warrants further investigation.

The role of integrin αV/TGF-β signaling in mediating OA progression provides a unifying explanation for the similar efficacy of the two interventions. DMM-induced joint instability generates abnormal mechanical stresses that activate αV integrins on chondrocytes and in subchondral bone. This leads to excessive TGF-β activation and Smad signaling (Zhen et al., 2021), which in turn drives production of degradative enzymes like MMP-13, as well as pro-inflammatory mediators (Werb et al., 1989; Arner and Tortorella, 1995; Homandberg, 1999; Gemba et al., 2002; Forsyth et al., 2002). In our untreated DMM mice, we observed exactly this molecular cascade: upregulated integrin αV, heightened p-Smad2/3, and increased MMP-13 in cartilage. Both swimming and hydrotherapy interrupted this pathological pathway. By reducing the intensity of mechanical stimuli on the joint, the interventions likely prevented abnormal integrin activation. Indeed, cartilage from both groups showed lower integrin αV levels and suppressed TGF-β signaling, along with a marked drop in MMP-13. MMP-13 is the principal collagenase responsible for collagen II cleavage in OA cartilage, even a partial reduction in MMP-13 can strongly protect cartilage structure. Therefore, it appears that a major benefit of both interventions is the mitigation of excessive integrin αV-mediated mechanotransduction. Swimming likely achieves this through dynamic unloading (the buoyancy of water reduces joint impact during movement), while hydrotherapy provides continuous unloading (no weight-bearing or shear forces at all) during immersion. The end result in both scenarios is reduced “wear and tear” signaling at the cell level, curbing the vicious cycle of cartilage damage.

Another important aspect of our study is the impact on skeletal muscle. Quadriceps weakness and atrophy commonly accompany knee OA and can exacerbate joint loading and instability. Exercise is known to counteract OA-related muscle loss by promoting muscle protein synthesis and strength. We found that swimming exercise indeed prevented quadriceps atrophy in DMM mice, as expected. The novel observation was that passive hydrotherapy preserved muscle mass to a similar extent. This outcome can be partly attributed to increased physical activity outside of therapy sessions–mice with less joint pain might move more in their cages, aiding muscle maintenance. However, there is likely a direct effect of the warm immersion on muscle physiology as well. Heat therapy has been shown to stimulate molecular pathways that maintain muscle fiber size, independent of exercise (Kim et al., 1985; Hyldahl and Peake, 1985). This finding is encouraging, as it suggests patients who cannot engage in vigorous exercise due to joint pain might still reap muscle benefits from simply soaking in warm water. Maintaining muscle mass is crucial for joint stabilization, and in our model, it presumably helped limit abnormal knee loads, complementing the direct cartilage benefits of hydrotherapy. Unfortunately, however, the specific mechanisms by which swimming or hydrotherapy improves quadriceps atrophy have not been elucidated. We examined the expression of selected integrins such as α7 and β1 that are highly correlated with muscle proliferation (Liu et al., 2008; Burkin and Kaufman, 1999) and found that swimming or hydrotherapy affected the expression of integrin β1 in the quadriceps muscle, which may be one of the mechanisms by which swimming or hydrotherapy improves quadriceps muscle atrophy, and the detailed process still needs to be further investigated.

The translational implications of these findings are significant. They suggest that passive hydrotherapy could be a viable low-impact therapy to slow OA progression, especially for individuals who are elderly, physically inactive, or unable to perform weight-bearing exercise. Hydrotherapy is already used empirically to manage arthritis symptoms (Lei et al., 2024; Lim et al., 2013), but our study provides scientific evidence of its disease-modifying potential on joint tissues. By demonstrating equivalent efficacy to swimming, we highlight that hydrotherapy is not merely palliative; it can directly influence the pathological mechanisms of OA. This could expand treatment options for patients who have contraindications to exercise or as an adjunct for those seeking additional relief. Hydrotherapy pools or even home baths maintained at therapeutic temperature might confer benefits if used regularly, as modeled in our twice-daily regimen. Clinically, combining hydrotherapy with a tailored exercise program might yield synergistic effects–for instance, a patient could begin with passive sessions to reduce pain and inflammation, then gradually add gentle exercises in water as tolerance improves. Indeed, clinical trials have reported that adding balneotherapy to conventional therapy results in superior outcomes in pain and function (Cantista and Maraver, 2020). Our findings lend biological plausibility to those results, showing that hydrotherapy favorably alters joint biology in much the same way as exercise.

While our study demonstrates compelling benefits of hydrotherapy in an animal model, some caution is warranted in extrapolating to humans. Mice have high metabolic rates and thin musculature, which may allow heat interventions to have pronounced effects; the response in human joints and muscles might differ. Moreover, our hydrotherapy protocol involved short, controlled sessions in warm water. This is distinctly different from prolonged immobilization, which over extended periods can lead to muscle weakening and cartilage thinning from disuse. Our results should therefore be interpreted as supporting intermittent passive therapy within a regimen that ultimately encourages activity. Future studies could explore the optimal balance between passive and active treatments and whether hydrotherapy’s benefits can be maintained long-term or during later stages of OA.

Mechanistically, there remain open questions about how exactly warm-water immersion exerts its chondroprotective effects. The present data point to reduced integrin αV activation as a major factor. At rest, intra-articular knee temperature is typically several degrees below core. Warm-water immersion may transiently elevate periarticular and possibly intra-articular temperatures, which could contribute beneficial effects alongside buoyant unloading. Reduced synovial-fluid viscosity at higher temperatures may facilitate lubrication, while modest heating can induce protective stress-response programs in chondrocytes *in vitro* (Becher et al., 2008). Our protocol used 37 °C water for both interventions, which is below cytotoxic thresholds reported for cartilage but sufficient to alter tissue thermodynamics and perfusion. We therefore interpret our data as consistent with a combined thermal and mechanical mechanism. Definitive partitioning of these components will require direct intra-articular temperature monitoring and temperature-controlled comparator arms. One hypothesis is that the hydrostatic pressure during water immersion provides a mild, uniform mechanical stimulus to chondrocytes. Chondrocytes can respond to static pressure by enhancing matrix synthesis within physiological ranges (Correia et al., 2012). Thus, gentle constant pressure might induce a benign mechanotransduction that favors anabolic activity without triggering the pathological αV/TGF-β pathway. However, this mechanism remains speculative, and the specific contribution of hydrostatic pressure *in vivo* is uncertain.

We focused on μCT morphometrics at 4 and 8 weeks, subtle subchondral bone remodeling below our detection threshold cannot be excluded, particularly at earlier timepoints. Future studies should test longer aquatic exposures and compare low-strain (aquatic) *versus* weight-bearing (treadmill/land-based) protocols to separate unloading-driven chondroprotection from osteogenic thresholds in subchondral bone. Additionally, the thermal effect increases blood flow and perfusion in periarticular tissues. Better circulation could aid in flushing out pro-inflammatory mediators from the joint and in delivering nutrients and oxygen to cartilage and bone. The heat may also induce protective chaperones in chondrocytes analogously to muscle, helping cells cope with stress. These hypotheses warrant further investigation. Identifying the precise cellular triggers (whether integrin, growth factor, or ion channel pathways) activated or inhibited by hydrotherapy will deepen our understanding of joint mechanobiology and guide optimized therapies.

It should be particularly noted that the absence of additional control groups at distinct water temperatures (such as colder or warmer conditions) may limit the ability to fully separate the thermal effects of hydrotherapy from the mechanical effects associated with buoyancy. However, hydrotherapy at 37 °C was intentional and has clinical relevance, as it closely mimics body temperature and is widely used in therapeutic aquatic interventions. On the one hand, previous guidelines indicate that water temperature significantly influences the swimming behavior of mice (Kregel et al., 2006). Typically, mice swim comfortably at temperatures close to their body temperature (approximately 36 °C) or room temperature (around 23 °C), whereas swimming below 23 °C may decrease core body temperature and swimming speed. On the other hand, the fundamental purpose of this research is clinical translation to patients with osteoarthritis, which predominantly affects middle-aged and elderly populations. Water temperature close to human body temperature may improve comfort and compliance among these patients during hydrotherapy interventions. Consequently, we specifically selected 37 °C water for both swimming and hydrotherapy groups. Moreover, the primary objective of our study was to evaluate the physiological and biomechanical impact of hydrotherapy and swimming, not to study temperature-dependent effects. Previous studies have highlighted the role of body-temperature water in enhancing circulation, reducing joint stress, and promoting muscle relaxation and therefore, underscore the clinical value of warm-water interventions for musculoskeletal and functional rehabilitation (Verhagen et al., 2012; Mooventhan et al., 2014; Wang et al., 2023). Nonetheless, we must acknowledge that the lack of temperature control conditions remains a limitation of this study. Future studies could incorporate additional temperature controls to better isolate and elucidate the temperature-specific effects of hydrotherapy interventions. In addition, we did not instrument animals for home-cage activity tracking, and thus cannot fully exclude between-group differences in spontaneous activity as a contributing factor. Nevertheless, identical housing, feeding, handling, schedule, and water temperature across intervention groups, together with convergent attenuation of αV/TGF-β signaling in both swimming and hydrotherapy, argue that the aquatic exposure itself was the principal driver of the observed molecular changes. Definitive causal tests will be required in future studies.

In summary, our study provides evidence that passive warm-water hydrotherapy can serve as an effective therapeutic intervention in knee osteoarthritis, producing outcomes on par with traditional exercise in a preclinical model. Both active swimming and static immersion curtailed the pathological remodeling of cartilage following DMM injury, potentially by modulating aberrant mechanotransductive signaling via the integrin αV/TGF-β pathway.

The preservation of quadriceps muscle in both groups further underscores the multifaceted benefits of the aquatic environment. These findings encourage a rethinking of rehabilitation strategies for OA–emphasizing that creating a favorable mechanical and thermal environment for the joint can be as impactful as active exercise in certain contexts. Ultimately, translating these insights to clinical practice could improve quality of life for OA patients. Hydrotherapy, a low-risk and accessible modality, might be particularly useful for patients with severe pain or mobility limitations, allowing them to receive the joint-protective “dose” of mechanical off-loading and thermal therapy necessary to slow disease progression. Further clinical studies are justified to test whether regular warm-water immersion can indeed modify structural disease outcomes in OA and to determine the optimal protocols (water temperature, session duration/frequency) for maximum benefit. If successful, passive aquatic therapy could become a valuable adjunct or alternative in OA management–harnessing the therapeutic power of water to ease the burden of this degenerative joint disease.

## Data Availability

The original contributions presented in the study are included in the article/Supplementary Material, further inquiries can be directed to the corresponding author.
